# A libraries reproducibility hackathon: connecting students to university research and testing the longevity of published code

**DOI:** 10.12688/f1000research.156917.2

**Published:** 2025-09-09

**Authors:** Chasz Griego, Kristen Scotti, Elizabeth Terveen, Joseph Chan, Daisy Sheng, Alfredo González-Espinoza, Christopher Warren

**Affiliations:** 1 University Libraries, Carnegie Mellon University, Pittsburgh, Pennsylvania, 15213, USA; 2 Carnegie Mellon University School of Computer Science, Pittsburgh, Pennsylvania, 15213, USA; 3 Carnegie Mellon University Department of English, Pittsburgh, Pennsylvania, 15213, USA

**Keywords:** Reproducibility, Hackathon, Academic Libraries, Open Science, Metascience, Digital Humanities, Computational Research, Software, Community Engagement

## Abstract

**Background:**

Reproducibility is a basis of scientific integrity, yet it remains a significant challenge across disciplines in computational science. This reproducibility crisis is now being met with an Open Science movement, which has risen to prominence within the scientific community and academic libraries especially. At the Carnegie Mellon University Libraries, the Open Science and Data Collaborations (OSDC) Program promotes Open Science practices with resources, services, and events. Hosting hackathons in academic libraries may show promise for furthering such efforts.

**Methods:**

To address the need for reproducible computational research and promote Open Science within the community, members of the OSDC Program organized a single-day hackathon centered around reproducibility. Partnering with a faculty researcher in English and Digital Humanities, we invited community members to reuse Python code and data from a research publication deposited to Harvard Dataverse. We also published these materials as a compute capsule in Code Ocean that participants could also access. Additionally, we investigated ways to use ChatGPT to troubleshoot errors from rerunning this code.

**Results:**

Three students from the School of Computer Science participated in this hackathon. Accessing materials from Harvard Dataverse, these students found success reproducing most of the data visualizations, but they required some manual setup and modifications to address depreciated libraries used in the code. Alternatively, we found Code Ocean to be a highly accessible option, free from depreciation risk. Last, ChatGPT also aided in finding and addressing the same roadblocks to successfully reproduce the same figures as the participating students.

**Conclusions:**

This hackathon allowed several students an opportunity to interact with and evaluate real research outputs, testing the reproducibility of computational data analyses. Partnering with faculty opened opportunities to improve open research materials. This case study outlines one approach for other academic libraries to highlight challenges that face reproducibility in an interactive setting.

## Introduction

Reproducibility in scientific research is a highly regarded concept that measures both the credibility and soundness of rigorous studies. ^1, 2^ In the context of research, the term reproducibility is often loosely defined or used interchangeably with replicability. The National Academy of Sciences convened in 2019 to develop a definition ^3^ and differentiate between reproducibility, replicability, and generalizability. ^2, 3^ Reproducibility, which is generally consistent with *computational* reproducibility, is defined as consistent computational outcomes by utilizing identical input data, procedures, methods, code, and analytical conditions. Replicability is defined as uniform outcomes across research projects designed to address the same scientific question, where each project collects its own data. And finally, generalizability is defined as how well findings of a study can be applied to different contexts or populations beyond the original.

Modern research continues to face multiple challenges surrounding reproducibility. The adoptable skills and practices that promote reproducibility may vary between fields, ^4– 6^ awareness or guidance is often lacking, ^4, 6, 7^ and the time and resources devoted to teaching and practicing reproducibility may be deficient. ^8^ Computation research, in particular, faces barriers to reproducibility ^9^ with software inconsistencies, changing versions and dependencies, ^10^ and insufficient documentation of datasets ^11^ or methods. ^12^ For example, Liu and Salganik ^11^ found that the complexity of employed computational methods impacts reproducibility. The more common methods in a given field may ease reproducibility, as they are familiar and well-documented, but more advanced methods can pose greater challenges, requiring specialized knowledge, environments, and resources that are not as readily available or standardized.

Investing in tools and practices around computational reproducibility enhances the transparency and reliability of scientific findings by enabling independent verification of results and fostering further research on established findings. Such a shift in computational research practice is important since it increases research credibility and efficiency and avoids wasted efforts in attempting to build on unreliable results. ^13, 14^ The scientific community is increasingly advocating for the use of various tools and practices aimed at improving reproducibility of computational research, ^12, 15^ including adopting open-source software, ^12^^,^^16^ conducting version control (e.g., Git and GitHub), ^17^ encapsulating computing environments (e.g., Docker and Guix), ^12, 18, 19^ and following community-driven standards and frameworks. Reproducible journals like Image Processing On Line (IPOL) ^20^ and repositories like Zenodo and Software Heritage are further supporting this shifting practice, allowing further sharing and archiving of research software and source code.

In general, scientific communities are upping advocacy through the Open Science movement. ^21^ Open Science promotes transparency of research process, data, methodologies, and outputs; enhancing accessibility and usability by the broader scientific community. ^22, 23^ Reproducibility is fundamental to open science, ^23, 24^ by ensuring verifiable scientific claims to the community; made possible via open sharing of datasets, methodology, code, and using open source software. Overall, alignment with reproducible practices moves the scientific community towards a more collaborative environment. ^22^

At the Carnegie Mellon University Libraries, a dedicated Open Science & Data Collaborations Program (OSDC) was established in 2018, which highlights the Libraries’ resources and tools that foster open, transparent, and reproducible research. ^25^ In this program, librarians and functional specialists serve as advocates, consultants, and collaborators for researchers accessing the existing Open Science infrastructure in the Libraries, including CMU’s institutional repository, KiltHub, ^26^ and research data management services. ^27^ In addition, the Libraries have held multiple iterations of the Open Science Symposium, ^28^ which gathered global researchers and thought leaders in academia, industry, and publishing to discuss the ways that Open Science has transformed research. The OSDC program also created roles for STEM PhDs to transition into Open Science and librarianship through an Open Science Postdoctoral Associate position, with Chasz Griego and Kristen Scotti being the first and second candidates in this role, respectively. Through this postdoctoral role, Griego considered ways to explore reproducibility integration and evaluation among researchers and students at CMU. ^29^

Motivated by the challenges surrounding reproducibility and the growing awareness of Open Science, members of the CMU Libraries and OSDC Program organized a single-day hackathon centered around reproducibility. Hackathons, which are time-bound events for groups to collectively solve or explore a technical problem, often find libraries as a suitable host as a neutral and welcoming space on campus that harbors learning, discovery, and collaboration. ^30^ The CMU Libraries has effectively hosted multiple hackathons, including Biohackathons and a recent AI Literacy Resource Hackathon. ^31– 33^

Libraries and hackathons surrounding reproducibility is also not a novel concept. The Center for Digital Scholarship at Leiden University held ReprohackNL in 2019, where participants attempted to reproduce published results using authors’ provided data and code in a single day event. ^34^

At the ReprohackNL event, the organizers observed how participants faced challenges with insufficient documentation, data behind a paywall, and problems with code and proprietary software. The CMU Libraries Reproducibility Hackathon aimed to offer a similar model, but here, we focused on the reproducibility of a study with shared data and code around a subject that could create interdisciplinary collaboration across students and researchers at CMU.

## Purpose of the event

The CMU Libraries Reproducibility Hackathon was an event that allowed any participant the opportunity to reproduce research results produced by a university professor. The purpose of such an event is to increase awareness of reproducibility as a realistic piece of the research life cycle, and demonstrate the outcomes of research scrutiny, which could theoretically be done by anyone. Most are familiar with the concept of reproducibility at a surface level, but many may not actually have a grasp on what that may look like, how it may happen, or how impactful it could be for any study. In an extremely idealistic world, a person with any background, experience, or research interest would have access to every material behind a particular study. These materials, again in an ideal case, would include all resources and guidance for a person to reproduce each result, assuring the findings first-hand. In reality, most research is very distanced from such an idealistic scenario, however, an event like a reproducibility hackathon can actually attempt to measure this distance for researchers willing to participate. For this hackathon, we offered one academic researcher the opportunity to put their research outputs up to scrutiny. Regardless of the outcome, a volunteer researcher helps set an example. If the results are highly reproducible, this presents a recognizable feat, and if the results prove more difficult to reproduce, this presents an opportunity to reflect on ways to improve. Work that is less reproducible does not have to be met with shame or guilt, but a humbling opportunity to learn and do better.

For the CMU Libraries Reproducibility Hackathon, we teamed up with Christopher Warren, a Professor of English and Associate Department Head in the Dietrich College of Humanities and Social Sciences at CMU. As the subject for a reproducibility assessment, Warren offered the content of a published data analysis of the Oxford Dictionary of National Biography (ODNB), ^35, 36^ a collection of biographies for over 60,000 influential figures in British history. Warren’s work is credited as an “audit” of the ODNB, ^37^ revealing the biases and assumptions hidden beneath the vastness of big data infrastructure. This subject was well aligned with the event, because with an analysis such as Warren’s, which scrutinizes the soundness of a massive data collection, the Python code that reveals the findings must also hold up to its own audit, ensuring that changing versions and dependencies do not prevent repeat analyses. With Warren offering his humanities research as an exemplary case, we offer further dialog around inclusivity of all research areas beyond STEM in the Open Science movement. ^38^

## Event execution

We openly distributed a call for participants to anyone interested from CMU and other institutions in the greater Pittsburgh area. The call noted that knowledge and awareness of scientific programming with Python and/or R was a desired, but not required, condition for participation. Participants submitted their interest and background through a short form and had the option to attend one of two information sessions a week prior to the event. During the information session, participants received a link to Warren’s manuscript to provide sufficient time to read into the details of the work. They were asked to not search for any items related to the work, avoiding the risk of early access to data and code posted to a repository.

**Figure 1.**
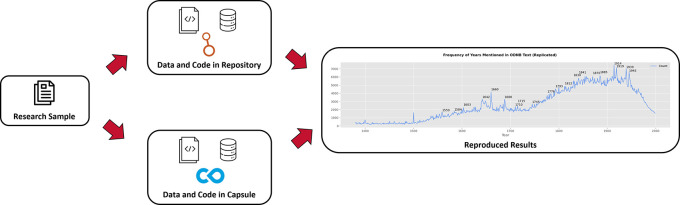
Diagram depicting the two options to reproduce research during the hackathon. The first option (top) uses research outputs stored in Harvard Dataverse, and the second option (bottom) uses a capsule in Code Ocean. The end of the diagram (right) shows a reproduced version of Figure 1 from Warren’s work. ^31^ Icons attributed to the Noun Project, the Dataverse Project, and Code Ocean.

The hackathon was scheduled for just one day, with participants and organizers convening in a library space during typical business hours (9am - 5pm). A project page on Open Science Framework (OSF) ^39^ was created for the event, where participants could access event information and guidance, a copy of Warren’s manuscript, and documents with links to access data and code. In addition to the published manuscript, Warren deposited supplementary research outputs to Harvard Dataverse, which includes a Jupyter Notebook, CSV files, Open Refine transformation JSON files, and others. ^36^ This collection of outputs served as one option for participants to use to attempt to reproduce the study ( Figure 1). This option is arguably open, having research outputs deposited in a repository to ensure secure storage, public access, and findability with a DOI, and this option used non-proprietary file formats such as CSV and JSON, and open-source Python code written in notebooks. Such attributes greatly promote reusability once users download each output and ensure that Python and all necessary libraries are installed.

We also provided a second option that aims to greatly facilitate reproducible computational research ( Figure 1). The same data and code found in Harvard Dataverse were organized and configured in a Code Ocean capsule. ^35^^,^^40^ Code Ocean is a platform that allows researchers to archive research data and code along with preserved language and library versions plus dependencies and a metadata record in a self-contained capsule. Capsules are discoverable with a DOI, and outside users can perform reproducible runs that compile and execute code in a virtual environment, producing results that match the original analysis.

## Observations and reflections

The reproducibility hackathon hosted three undergraduate participants, who were each in the first or second year of their degree program in the School of Computer Science with established Python programming experience. Each student is an author of this work, with the following summarizing the notable outcomes, experiences, and contributions of these participants. Some notes are also listed briefly in Table 1. One participant, Elizabeth Terveen, chose the option of downloading the materials from Harvard Dataverse and attempting to run the Jupyter Notebook in a new Python environment working with the Anaconda package manager. To run the code, Terveen had to install four Python libraries (Pandas, Nltk, Matplotlib, and Plotly). One of the existing import statements in the notebook, regarding Plotly, returned an error due to a module depreciation.

**Table 1.** Technical issues experienced from participants attempting to reproduce results by running the code corresponding to Warren’s work. ^31^

Notable issues
Several Python libraries required installation
Some import statements needed to be updated because of module depreciation
New arguments were required to successfully run a function
A function keyword argument name was updated
File paths in the code were not consistent with file organization in the repository

Of the twenty-five figures produced in the notebook, Terveen noted fourteen figures that were successfully reproduced, but six of these fourteen figures did require some troubleshooting. This included supplying values for arguments in functions that were not specified in the original code, but a resulting error stated as required. The name of a keyword argument related to a Matplotlib function had also become depreciated, which required an update to the original code. Eight figures were not successfully reproduced because of file paths in the code that were not consistent with directory structure Terveen had after downloading from the repository. The reproducibility of two figures, however, werewas not documented during the event.

A second participant, Joseph Chan, also chose the option to download the materials from Harvard Dataverse. Chan immediately noted that if a “requirements.txt” file was included with the original deposit, any user trying to reproduce the results would be able to download and install the appropriate packages and versions. Based on Chan’s setup, five python libraries and Jupyter needed to be installed, including Pandas, Nltk, Pickle, Numpy, and Matplotlib. Additionally, a plotting library was deprecated, requiring an import statement to be updated. Once again, keyword arguments were depreciated and had to be updated. Two figures did not display upon running and were unable to be solved.

Chan also created a modified version of the research outputs as a zip file uploaded to OSF ( https://osf.io/hvbzm), which contains a requirements.txt file, all necessary data, and all the code from the notebook in a single Python (.py) file. With this, users can simply reproduce the work by executing a shell script from a terminal. Copies of figures that were successfully reproduced were uploaded to OSF ( https://osf.io/gqk8e/), with titles altered with either a note or a name to verify that the figures were original copies (see Figure 1).

Overall, this process of reproducing results using files downloaded from a repository produced several challenges with requirements to install packages, understand depreciations for some packages, and troubleshooting changes in syntax from evolving versions of software. Though there was trial and error, participants were able to reproduce a majority of the figures from this study. Additionally, participants provided their own insight to develop an updated collection of research outputs that help others reproduce this work more easily.

## Additional outcomes and lessons learned

Alfredo González-Espinoza, our colleague who participated in the assessment as well, offered an interesting anecdote while pursuing reproducibility via both Harvard Dataverse and Code Ocean ( https://osf.io/brn6d). In order to run the code downloaded from the repository, he had to install Python, Jupyter, all required libraries, and found the same depreciation errors as other participants; however, he was successful with reproducibility via the Code Ocean capsule. González-Espinoza’s scenario was interesting because he used a gaming laptop to reproduce from the repository, which doesn’t make a difference when the issue is installing software, but for the capsule he accessed the code via an old Chromebook tablet. This individual experience calls to attention the fact that a platform like Code Ocean may promote accessibility for people that may have limited access to computers, and instead only have access to devices that one can’t use to download and install software as easily.

The hackathon offered an additional opportunity for exploration. Kristen Scotti investigated ways to troubleshoot reproducing the output of the code using generative AI. Python, like many other programming languages, undergoes frequent updates, deprecating functions or changing syntax overtime, making author-provided code more difficult to work with or potentially incompatible with newer versions. Here, Scotti ran the author-provided code through ChatGPT to request updates ( https://osf.io/r65ev). ChatGPT’s programming capabilities include the ability to interpret, debug, and suggest updates to code. ^41^ ChatGPT updated code successfully for all recreated plots except one, which also provided issues for other participants. The likely culprit was formatting issues due to syntax changes and complexity of the provided code block. A more effective approach might be to ask ChatGPT to convert the code in smaller sections, though this often requires someone experienced in the language to fine-tune both the prompts and the outputs.

**Figure 2.**
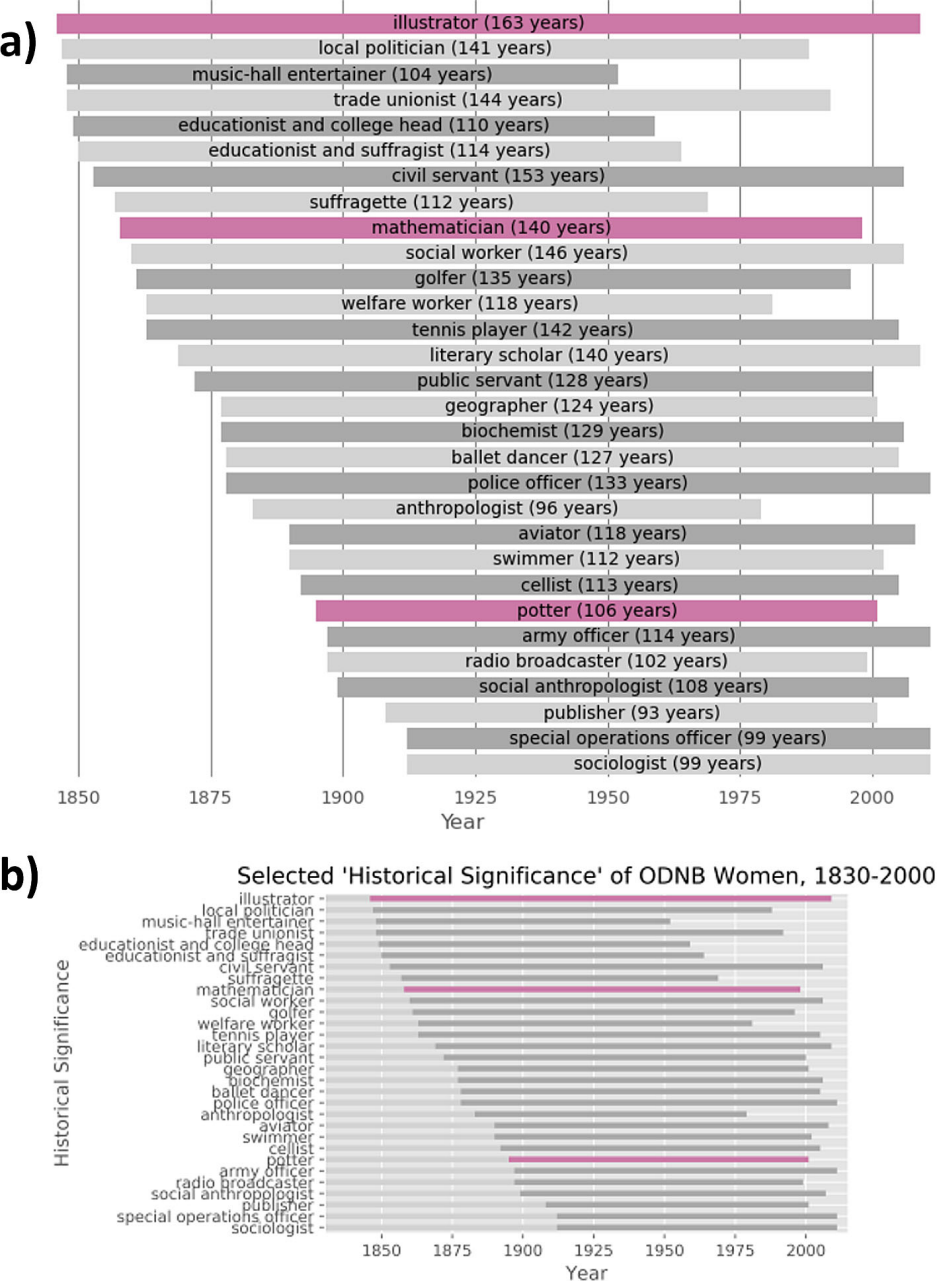
Adapted version (a) of Figure 29 (b) from Warren’s work. ^31^

Outside the hackathon setting, we revisited the capsule version of Warren’s research to test how easily his work could be adapted within this platform. For instance, figures 28 and 29 in the manuscript have prompted discussion in other sources. ^37, 42^ Figure 29, in particular, displays the historical significance of selected women in the ODNB, and the span of living years of these women from 1830-2000 (listed here in Figure 2b). However, horizontal bars representing a span of years could instead incidentally suggest the number of women belonging to the corresponding historical significance. We addressed this ambiguity by creating a new version of the capsule on Code Ocean ^43^ where additional cells in the Jupyter Notebook create new versions of these figures which explicitly state the numbers of years each vertical bar spans ( Figure 2a).

## Discussion and conclusions

We have outlined the execution and outcomes of a Reproducibility Hackathon, hosted by CMU Libraries, focusing on the reusability of data and code shared by a faculty member from the university. This event allowed students to interact with real research outputs to learn potentially new skills and fields of study while simultaneously offering their perspective. In reviewing the reproducibility of research code from the participating faculty member, Christopher Warren, students showed that manual setup was needed to rerun research code, and along with that, depreciations required them to write updates and modifications. One participating student sought a way to encapsulate the code so that everything was packaged and shareable, enabling an outside user to properly reproduce the run by executing a single script. Lastly, we attempted to rerun the code while using a ChatGPT API to debug. This option identified and addressed the same errors that student participants met and successfully reproduced the same figures.

The Reproducibility Hackathon presented just one of countless cases of research code that won’t rerun reliable as it. Python code and its many libraries for scientific analysis will update regularly, where over the years, significant changes like those we found will likely happen. Documentation is important to circumvent these changes to code so that external users are confidently aware of the original file organization and hierarchy, versions and dependencies of software libraries, and even potentially the original operating system or computing hardware. Though even with all details noted, an external user may lack all of the resources necessary to fully reproduce the analysis, but the combination of open source licensing, thorough documentation, and full transparency gives these users confident starting points to further research progress.

Code Ocean presented an alternative to sharing code without manual installations or depreciations. An alternative of Warren’s outputs was assembled and published in a reproducible capsule on Code Ocean. This option presented itself as a more accessible way for a user to interact with computational research, as a participating colleague revealed the ease of interacting with the capsule on a tablet device. Further applying this option, we modified Warren’s code in Code Ocean to produce an alternative depiction of data visualizations presented in the original work. Doing this offered an example of how a reproducibility-centered platform can promote research as ongoing conversation that openly invites community members to offer constructive insights.

In addition to Code Ocean, we also promoted the usage of Open Science Framework (OSF) throughout the Reproducibility Hackathon. Both platforms did greatly facilitate access of research documents, data, and code, but we also highlight that these platforms invoke terms of use related to the research data, software, and other content that is stored and manipulated within them. These terms, or the nature of sharing research material on these platforms from such companies, may not always align with the goals or values of individual researchers, teams, or institutions, and discretion is always recommended. These platforms are just a few of the numerous options, including institutional platforms and services, available to researchers to share open and reproducible outputs.

Hosting a reproducibility hackathon at an academic institution created an open environment for researchers to put their published work to the test, opening themselves to constructive feedback around reproducibility, and allowing students or other researchers to interact with real research artifacts. The CMU Libraries reproducibility hackathon allowed three undergraduate students from the School of Computer Science to interact with a unique study in Digital Humanities. This was particularly meaningful because these students may not have otherwise had the opportunity to interact with real research in this way and see examples of the work done in this discipline. Collaborating with a faculty researcher, like Warren, further sets examples of researchers who not only open up research materials, but embraced opportunities to revisit and improve their work to maintain its relevance and scholarly integrity.

We initially aimed for a higher number of participants, but we only received interest from eight students, with three being the listed participants here. For future iterations of this event, we will consider other factors to gain interest and retention. The time commitment of a full day may be excessive, considering class schedules. A shorter event, hosted after most classes in the evening could promote higher participation. Furthermore, the subject matter of the research may not be widely interesting across campus, suggesting a more targeted form of marketing, either to individual academic departments or courses. While we stated several benefits from our collaboration with Warren, other interested faculty researchers may have varying abilities to commit for subsequent events at any given time. While our execution was not one-size-fits-all, nor optimum, we have presented our experience here to offer a blueprint for other academic libraries to execute similar events, boost awareness of the threats to preserving research code, and highlight how new platforms eliminate these threats to reproducibility and enhance adaptation along the research lifecycle.

## Ethics and consent

Ethical approval and consent were not required.

## Author contributions

**Chasz G:** Conceptualization, Data Curation, Formal Analysis, Methodology, Project Administration, Resources, Software, Supervision, Validation, Writing – Original Draft Preparation; **Kristen S:** Conceptualization, Data Curation, Formal Analysis, Resources, Software, Validation, Visualization, Writing – Original Draft Preparation; **Elizabeth T:** Data Curation, Formal Analysis, Validation, Visualization; **Joseph C:** Data Curation, Formal Analysis, Software, Validation; **Daisy S:** Data Curation, Visualization; **Alfredo G-E:** Data Curation, Formal Analysis, Validation; **Christopher W:** Conceptualization, Data Curation, Resources, Software, Visualization, Writing – Review & Editing

## Data Availability

Open Science Framework: Reproducibility Hackathon 2024. https://doi.org/10.17605/OSF.IO/AQ69R. ^39^ The project contains the following underlying data:
• All original documents and materials prepared and produced from the event. Data are available under the terms of the Creative Commons Attribution 4.0 International license (CC-BY 4.0). Harvard Dataverse: Replication Data for: Historiography’s Two Voices: Data Infrastructure and History at Scale in the Oxford Dictionary of National Biography (ODNB). https://doi.org/10.7910/DVN/D3KFLP. ^40^ The project contains the following underlying data:
• All data and code supporting the original work that was the focus of the hackathon. Data are available under the terms of the Creative Commons 1.0 Universal Dead (CC0 1.0) Code Ocean: Replication Data for: Historiography’s Two Voices: Data Infrastructure and History at Scale in the Oxford Dictionary of National Biography (ODNB) (Version 1). https://doi.org/10.24433/CO.6313661.v1. ^35^ The project contains the following underlying data:
• All data, code, and software environment details supporting the original work that was the focus of the hackathon. Data and code are available under the terms of the Creative Commons CC0 licenses with no rights reserved. Code Ocean: Replication Data for: Historiography’s Two Voices: Data Infrastructure and History at Scale in the Oxford Dictionary of National Biography (ODNB) (Version 2). https://doi.org/10.24433/CO.6313661.v2. ^43^ The project contains the following underlying data:
• All data, code, and software environment details supporting the original work that was the focus of the hackathon as well as appended code that adapts Figures 28 and 29 from the original study. Data and code are available under the terms of the Creative Commons CC0 1.0 licenses with no rights reserved.
