# Supplementary material for: A libraries reproducibility hackathon: connecting students to university research and testing the longevity of published code
Source: F1000Res. 2025 Sep 9;13:1305. Originally published 2024 Oct 31. [Version 2] doi: 10.12688/f1000research.156917.2 (PMC12501581; doi:10.12688/f1000research.156917.2)
Supplement: Peer review history

# Reviewer report: Reviewer response for version 2

**DOI:** 10.5256/f1000research.187759.r413599

**Published:** 2025-10-15

**Authors:** Stephen J Eglen

**Affiliations:** 1 Cambridge University, Cambridge, UK

As this paper has already been through review, it looks like most of the key points have already been noted and corrected.

 I found the study easy to read and am supportive of indexing. I have just a few minor suggestions that might improve the paper.

 "Eight figures were not successfully reproduced because of file paths in the code": could you fix the code, or did the author get feedback about what went wrong here?

 "were was not documented" 

 Was the author present when you did the hackathon? (Our experience in codecheck hackathons is that they go much quicker, as expected, when authors are present to address any issues.)

 Thank you for providing the supplementary information that shows what you did at the event; could you possibly annotate (e.g. a README) for your osf.io collection so that people can easily see what you have shared?  It wasn't immediately clear to me what you had provided.

 As a general comment, if you were to run another hackathon along these lines, is there anything that you would do differently next time?

Is the case presented with sufficient detail to be useful for teaching or other practitioners?

Partly

Is the work clearly and accurately presented and does it cite the current literature?

Yes

If applicable, is the statistical analysis and its interpretation appropriate?

Yes

Are all the source data underlying the results available to ensure full reproducibility?

Yes

Are the conclusions drawn adequately supported by the results?

Yes

Is the background of the case’s history and progression described in sufficient detail?

Yes

Reviewer Expertise:

Computational Neuroscience; Reproducible Research (codecheck.org.uk)

I confirm that I have read this submission and believe that I have an appropriate level of expertise to confirm that it is of an acceptable scientific standard.

# Reviewer report: Reviewer response for version 2

**DOI:** 10.5256/f1000research.187759.r412602

**Published:** 2025-10-06

**Authors:** Teresa Gomez-Diaz

**Affiliations:** 1 Universite Gustave Eiffel / Laboratoire d'informatique Gaspard-Monge / CNRS, Marne-la-Vallée, Île-de-France, France

The second version is fine. The authors have replied to all comments, and they have fixed the main issues. This work relates a very interesting reproducibility experience that took place at the Carnegie Mellon University Libraries.

Is the case presented with sufficient detail to be useful for teaching or other practitioners?

Partly

Is the work clearly and accurately presented and does it cite the current literature?

Partly

If applicable, is the statistical analysis and its interpretation appropriate?

Partly

Are all the source data underlying the results available to ensure full reproducibility?

Yes

Are the conclusions drawn adequately supported by the results?

Partly

Is the background of the case’s history and progression described in sufficient detail?

Partly

Reviewer Expertise:

Research Software, Research Data, Open Science, dissemination, evaluation

I confirm that I have read this submission and believe that I have an appropriate level of expertise to confirm that it is of an acceptable scientific standard.

# Reviewer report: Reviewer response for version 1

**DOI:** 10.5256/f1000research.172297.r355235

**Published:** 2025-01-07

**Authors:** Teresa Gomez-Diaz, Tomas Recio

**Affiliations:** 1 Universite Gustave Eiffel / Laboratoire d'informatique Gaspard-Monge / CNRS, Marne-la-Vallée, Île-de-France, France; 2 Community of Madrid, Universidad Antonio de Nebrija, Madrid, Spain

CASE STUDY

 A libraries reproducibility hackathon: connecting students to University research and testing the longevity of published code https://f1000research.com/articles/13-1305/v1

 This case study refers to a hackathon conducted by the members of the Carnegie Mellon University (CMU) Libraries in order to reproduce research results produced by Professor Christopher Warren on data analysis of the Oxford Dictionary of National Biography (ODNB). The event was open for participants coming from CMU and other institutions in the greater Pittsburgh area and was conducted by members of the CMU Libraries. This experience gives an interesting setting to explore reproducibility of published research results by a Professor of the University who also provides data and the produced software involved in the research, and shows a good example of a collaboration between Librarians and members (students?) of the Computer Science School.

 However, there are a few points in this manuscript that need attention.

 1. Abstract. As included in the Case Studies guidelines [ https://f1000research.com/for-authors/article-guidelines/case-studies ], abstract should be structured into **Background, Methods, Results,** and **Conclusions** . In particular, the conclusions should be better presented in the abstract (and in the manuscript, see later).

 2. Hackathon context. As explained in the document (see also [ https://www.library.cmu.edu/abou)t/news/2024-04/reproducibility-hackathon ], the event was open to a large (institutional) public, but the title of the manuscript refers to students. More insight about the participants (how many, how many students, which level, which scientific background…) should be provided in order to better seize the complexity of the event. Please, clarify if the three authors of the manuscript coming from the Computer Science School where also participants of the event and if they are students or Members of the School. Moreover, it was indicated that “participants could have knowledge and awareness of scientific programming with Python and/or R” was desired, but was not required. Was this a difficulty? How many participants had or had not programming knowledge?

 3. Problems with data and code It is mentioned that: “At this hackathon event, the organizers observed how participants faced challenges with insufficient documentation, data behind a paywall, and problems with code and proprietary software.” But there is no further information about these observations.

 4. Reproducibility success. It is mentioned that: “Of the figures produced in the notebook, fourteen figures were successfully reproduced by Terveen … and eight figures were not successfully reproduced.” Or that “Though there was trial and error, participants were able to successfully reproduce figures from this study”. But which is the percentage of success? How many figures were in the initial work by Prof. Warren and how many were reproduced by all the participants?

 5. Insufficient documentation and code longevity. As noticed by participant Chan, a “requirements.txt” file could help to facilitate the understanding of the software context of the code produced by Prof. Warren. Our work (see Gomez-Diaz T, et al., 2022 [Ref 1]) goes further in this kind of recommendations, proposing flexible procedures for research software and research data dissemination.

 It is extremely idealistic to expect that software released at some time will find easily runable environments, unless there is the available resources (human, funding) to keep the necessary maintenance of the produced software (who can use Windows version 5?). To accompany the research produced software with good documentation and description of the necessary components will help to get to reproducibility environments, that is, to follow appropriate dissemination procedures will be of help for reproducibility issues. Nevertheless, it will be not always possible to ensure the full reproducibility and, in our opinion, a partial solution consists in to release the research software as free/open source software, where the accessibility and the study of the source code is confirmed by the use of free/open source licenses.

 6. Conclusions. Conclusions are poorly described, and more information on the benefits, found drawbacks and other consequences of the event, for participants, students, Prof. Warren and event organizers should be provided to complete this case study.

 7. References. We would like to suggest the inclusion of these references:

- for Open Science: UNESCO: UNESCO Recommendation on Open Science. 2021 [Ref 2]
- for Free/Open Source Software: K. Fogel (2005-2022). Producing Open Source Software. How to Run a Successful Free Software Project, https://producingoss.com
- for an example of a Journal giving a reproducibility environment for their publications:
- IPOL Journal · Image Processing On Line [ https://www.ipol.im/
- Nicolaï, A., et al., 2022 [Ref 3]

8. Use of privately owned companies. We would like to raise awareness about the use of privately owned companies like Code Ocean [ https://codeocean.com/ ] for the storage and manipulation of data and code produced within the scientific community. Usually the scientific community is not well aware of legal issues related to the terms of use of these platforms and their consequences, like, for example: `` *You hereby grant Code Ocean a worldwide, perpetual, irrevocable, royalty-free right and license to use and exploit any ideas, suggestions, comments, recommendations, enhancement requests or other input provided by you about the Platform to Code Ocean.* '' (see point 3. **Feedback** in [ https://codeocean.com/terms-of-use ]). Regarding OSF, the Center for Open Science, Inc. (referred to as "the COS", "Open Science Framework", "OSF") declares to be a nonprofit organization, but also provides with similar terms of use: `` *by depositing any Content (including data and links, but not the linked content) in a COS account, you grant the COS a perpetual, irrevocable, worldwide, royalty-free license to store, reproduce, transmit, distribute, publicly perform, and publicly display such Happy…* '' (see further information in [ https://github.com/CenterForOpenScience/cos.io/blob/master/TERMS_OF_USE.md ]). Universities and other Research Performing Institutions, and, in particular, University and other institutional Libraries, could study the proposition of University or institutionally owned platforms and/or services to deal with their scientific production.

 9. To complete the background context of this case study, some information on previously hosted hackathons by the CMU Libraries, like Biohackathons and an AI Literacy Resource Hackathon, could be included as they can be of interest for the Hackathon community.

Is the case presented with sufficient detail to be useful for teaching or other practitioners?

Partly

Is the work clearly and accurately presented and does it cite the current literature?

Partly

If applicable, is the statistical analysis and its interpretation appropriate?

Partly

Are all the source data underlying the results available to ensure full reproducibility?

Yes

Are the conclusions drawn adequately supported by the results?

Partly

Is the background of the case’s history and progression described in sufficient detail?

Partly

Reviewer Expertise:

Research Software, Research Data, Open Science, dissemination, evaluation

We confirm that we have read this submission and believe that we have an appropriate level of expertise to confirm that it is of an acceptable scientific standard, however we have significant reservations, as outlined above.

## References

[1] : Research Software vs. Research Data II: Protocols for Research Data dissemination and evaluation in the Open Science context. *F1000Res* .2022;11: 10.12688/f1000research.78459.2 117 10.12688/f1000research.78459.2 36483317 PMC9706143

[2] : UNESCO Recommendation on Open Science.2021; 10.54677/MNMH8546 10.54677/MNMH8546

[3] : The approach to reproducible research of the Image Processing On Line (IPOL) journal. *http://www.scielo.edu.uy/pdf/info/v27n1/2301-1378-info-27-01-76.pdf* .2022;

# Response

**Published:** 2025-09-04

**Authors:** Chasz Griego

Thank you, we really appreciate your review and recommendations. We are grateful that you could offer your experience and perspective to help strengthen our paper, as well as offer questions that helped us add more clarity and critical points in our writing. Please see our responses to your comments, where we also note any changes made to the original manuscript:

*“abstract should be structured into Background, Methods, Results, and Conclusions . In particular, the conclusions should be better presented in the abstract (and in the manuscript, see later).”*

**Response: **Thank you for bringing this to our attention. We have restructured the abstract, resulting in something that we think will be easier for a reader to navigate. We also revised our conclusions, both here and later in the manuscript (also mentioned later in this response).

*“More insight about the participants (how many, how many students, which level, which scientific background…).... Please, clarify if the three authors of the manuscript coming from the Computer Science School where also participants of the event and if they are students or Members of the School.”*

**Response: **Thank you for noting this ambiguity. We've added a statement clarifying that the three authors from the School of Computer Science were undergraduate students participating in the event. This clarification did significantly improve our narrative and summary. 

*“it was indicated that “participants could have knowledge and awareness of scientific programming with Python and/or R” was desired, but was not required. Was this a difficulty? How many participants had or had not programming knowledge?”*

**Response: **This is good to clarify. Each student already had an established skillset with Python programming. With the limited pool of participants, a desire for programming knowledge was not a challenge, but this could be something to consider for future events, especially considering the aspect of reproducibility by any person or team.

*“It is mentioned that: “At this hackathon event, the organizers observed how participants faced challenges with insufficient documentation, data behind a paywall, and problems with code and proprietary software.” But there is no further information about these observations.”*

**Response: **We are grateful that you noted this because it actually revealed an error in our editing. This sentence is in reference to the cited hackathon in the previous statement (ReprohackNL). The paragraph break between these statements was an error. We fixed this error to hopefully make better sense.

*“But which is the percentage of success? How many figures were in the initial work by Prof. Warren and how many were reproduced by all the participants?”*

**Response: **Thank you for pointing out these details, which are a bit vague. We've edited the manuscript to mention that the original work had twenty-five figures to reproduce. We also changed the wording in the latter sentence to read with more accuracy. Our paper is not focusing on if the entire work was reproduced during this event, rather, the paper is focusing on the details and nuances of research code that are revealed when a group comes together to try to reproduce the outcomes.

*“Our work (see Gomez-Diaz T, et al., 2022 [Ref 1]) goes further in this kind of recommendations, proposing flexible procedures for research software and research data dissemination. It is extremely idealistic to expect that software released at some time will find easily runable environments, unless there is the available resources… Nevertheless, it will be not always possible to ensure the full reproducibility and, in our opinion, a partial solution consists in to release the research software as free/open source software, where the accessibility and the study of the source code is confirmed by the use of free/open source licenses.”*

**Response: **These were all excellent points, especially those written in your work. We decided to add another paragraph to the conclusion section to address how reproducibility also relies on adequate resources and systems, which their availability may also depreciate with time, and that documentation offers further guidance and transparency to help other users reuse research software and data, even when resources are scarce. We appreciated the opportunity to add this critical aspect to our conclusions. 

*“Conclusions are poorly described, and more information on the benefits, found drawbacks and other consequences of the event, for participants, students, Prof. Warren and event organizers should be provided to complete this case study.”*

**Response: **Thank you for this suggestion. Your review greatly helped us better communicate the impact and implications of running this type of hackathon. It also adds further context for readers to learn how to readapt this model. The final paragraphs of this paper now offer further detail about the benefits, drawbacks, and lessons learned.

*“We would like to suggest the inclusion of these references…”*

**Response: **Thank you for these great recommendations. These references were added to the appropriate places in the manuscript.

*“Use of privately owned companies…”*

**Response: **We found your point here one of the most important to call to attention. In our libraries, and even within our Open Science initiatives, we often promote tools and platforms citing the benefits of places that host your research materials and offer increased accessibility and interoperability. However, it is true that while these companies create these products, they also have business models to sustain and insurance around their products. This was important to note in our concluding section to highlight that researchers and librarians may have rightful reservations when using these products.

# Reviewer report: Reviewer response for version 1

**DOI:** 10.5256/f1000research.172297.r339316

**Published:** 2024-12-04

**Authors:** Benjamin Antunes

**Affiliations:** 1 Université Clermont Auvergne, Clermont-Ferrand, France

This paper presents a reproducibility hackathon where students attempted to replicate the work of Professor Christopher Warren. In my opinion, this is an excellent initiative. Education is a critical avenue for improving reproducibility, as it raises awareness among students and future researchers about its importance.

 The paper is not highly technical; instead, it provides an overview of the types of challenges encountered when attempting to reproduce results. It highlights the use of literate programming and ChatGPT to facilitate code reproduction and illustrates common difficulties, such as handling software dependencies and library versioning in Python. The hackathon focused on research in the field of Digital Humanities.

 My Remarks:

 Introduction – First Paragraph: As noted by Barba (2018), computer science was one of the only field to use swaped definitions between reproducibility and replicability (reproducibility is doing the same experiment to obtain the same results, while replicability is doing another experiment to obtain the same scientific conclusion). ACM recently adopted the NISO-recommended terminology for computer science:

 " *As a result of discussions with the National Information Standards Organization (NISO), it was recommended that ACM harmonize its terminology and definitions with those used in the broader scientific research community, and ACM agreed with NISO’s recommendation to swap the terms “reproducibility” and “replication” with the existing definitions used by ACM as part of its artifact review and badging initiative. ACM took action to update all prior badging to ensure consistency."

https://www.acm.org/publications/policies/artifact-review-and-badging-current

 This shift swaped the definition of reproducibility and replicability. However, from an epistemological standpoint, reproducibility remains the broader, general term.

 The paper omits a crucial concept in computer science: repeatability (bitwise repeatability), which is indispensable for debugging (in the context of computer science)

 Code execution depends on the entire software environment. Open-source software is essential, starting with the operating system (e.g., Linux). However, Docker might not be the best tool for encapsulating computing environments (in the context of reproducible research), as it was not initially designed for reproducibility. I suggest exploring Guix, which is purpose-built for reproducible research, and Software Heritage for archiving software.

 You rightly emphasize the importance of sharing code and data, aligning with the open science movement. It would be helpful to mention tools like Software Heritage and Zenodo for sharing resources. I noticed the mention of a locally managed public repository, KiltHub. If this repository is not accessible to researchers outside Carnegie Mellon University, highlighting alternative platforms like Zenodo remains relevant.

 The inclusion of Figure 1 might not add value to the reader. This paper is a practical feedback, primarily aimed at showcasing the challenges of reproducing work. Instead, figures illustrating reproducibility workflows or tools (e.g., Table 1) might be more informative.

 While Python is widely used due to its ease of use, it is not ideal for reproducibility because of its instability compared to languages like Bash or C, that are more stable. Nonetheless, I agree that results should be reproducible by all, including non-experts, so Python remains a good practical choice.

 The paper briefly mentions Docker in the introduction but does not elaborate on its usage. It may be more relevant to discuss tools like Guix and Software Heritage, which were explicitly created to address reproducibility challenges. While the paper focuses on Digital Humanities, tools such as CodeOcean (originating from bioinformatics) and FAIR principles are broadly applicable.

 The paper focus on Python Jupyter notebooks and literate programming is a solid first step for enabling reproducibility. However, other fields like biology and physics, among others, also face reproducibility crises. Broader discussions could include data storage, workflow tools (for example Snakemake to keep it simple), virtual machines, containers, floating-point arithmetic issues, or parallel computing. But of course, the objective of the paper is to present the hackathon, so we cannot talk about everything. I just write it here if you want to take a look.

 Computers, often seen as “black boxes,” create reproducibility challenges across disciplines. A short introduction to computer science and reproducible research should be incorporated into all scientific curricula. I can recommend you the MOOC: https://www.fun-mooc.fr/en/courses/reproducible-research-methodological-principles-transparent-scie/

 In addition, I have written one survey about reproducible research, even if it was more oriented to High performance computing, you might benefit from its read:

 Antunes B, et al., 2024 (Ref 1)

 One detail: In the PDF version of the paper, the abstract text is not justified. Shouldn’t it be?

 Conclusion

 This hackathon is an excellent initiative to raise awareness among students and young researchers about the challenges of reproducibility. While the paper provides a high-level overview, focusing on literate programming with Jupyter notebooks and the problem of software dependency hell, it serves as a good introduction to the topic.

Is the case presented with sufficient detail to be useful for teaching or other practitioners?

Yes

Is the work clearly and accurately presented and does it cite the current literature?

Yes

If applicable, is the statistical analysis and its interpretation appropriate?

Not applicable

Are all the source data underlying the results available to ensure full reproducibility?

Yes

Are the conclusions drawn adequately supported by the results?

Yes

Is the background of the case’s history and progression described in sufficient detail?

Yes

Reviewer Expertise:

My research area if reproducible research in high performance computing. This paper could benefit from having broader information about reproducibility tools, however, it is a short paper describing a Hackathon.

I confirm that I have read this submission and believe that I have an appropriate level of expertise to confirm that it is of an acceptable scientific standard.

## References

[1] : Reproducibility, Replicability and Repeatability: A survey of reproducible research with a focus on high performance computing. *Computer Science Review* .2024;53: 10.1016/j.cosrev.2024.100655 10.1016/j.cosrev.2024.100655

# Response

**Published:** 2025-09-04

**Authors:** Chasz Griego

Thank you for your review. We really appreciate your thoughtful assessment of our work, and we are thrilled to receive your perspective and recognition. Please see our responses to your comments, where we also note any changes made to the original manuscript:

*“ACM recently adopted the NISO-recommended terminology for computer science…”*

**Response:** We appreciate you noting the consistency of the definitions of reproducibility and replicability, between ACM (and NISO) with what is mentioned  in our paper, especially in the context of artifact badging. This was good to note, showing how there exists multiple guidelines that follow this convention.

*“The paper omits a crucial concept in computer science: repeatability (bitwise repeatability), which is indispensable for debugging (in the context of computer science)”*

**Response: **We definitely agree that bitwise repeatability is highly notable for fully reproducible computer science, and we're grateful that you note it here. However, as this paper is addressing a less technical scope of reproducible computational research (ie. basic data science and analysis, in contrast to high performance computing), we are content with the scope of detail on computational reproducible that we chose to mention.

*“It would be helpful to mention tools like Software Heritage and Zenodo for sharing resources. I noticed the mention of a locally managed public repository, KiltHub. If this repository is not accessible to researchers outside Carnegie Mellon University, highlighting alternative platforms like Zenodo remains relevant.”*

**Response: **Thank you for mentioning both of these platforms and suggesting we mention them. We agree and have included them in the third paragraph of the introduction, as they are greatly contributing to sharing and preserving software. We agree that a repository like Zenodo is a great alternative to an institutional repository, like KiltHub. However, we prefer to not highlight Zotero or other platforms in the paragraph with KiltHub, since the paragraph was designed to focus specifically on the background behind CMU's Open Science infrastructure, rather than the array of options available for open and reproducible research.

*“Instead, figures illustrating reproducibility workflows or tools (e.g., Table 1) might be more informative.”*

**Response: **This is a great suggestion. We instead decided to convert this figure into a diagram that illustrates the two options (paths) to reproducibility. This diagram still uses one of the original data visualizations as an example, but in much better context. 

*“The paper briefly mentions Docker in the introduction but does not elaborate on its usage. It may be more relevant to discuss tools like Guix and Software Heritage, which were explicitly created to address reproducibility challenges.”*

**Response: **Those are fair points. Again, we only mention tools/platforms and avoid adding excessive detail for the sake of the paper’s scope. However, we have now included mentions to Guix and Software Heritage based on your recommendation.

*“Broader discussions could include data storage, workflow tools (for example Snakemake to keep it simple), virtual machines, containers, floating-point arithmetic issues, or parallel computing.”*

**Response: **We really appreciate these points you mention. Reproducibility, let alone computational reproducibility, is so nuanced in each field. Issues surrounding data storage, high-throughput computing, and other software architectures are important to consider, and we hope that efforts like this hackathon, and conversations around it, will promote further awareness and practice that leads to these factors being further addressed.

*“I can recommend you the MOOC…. In addition, I have written one survey about reproducible research, even if it was more oriented to High performance computing, you might benefit from its read…”*

**Response: **Thank you for sharing the MOOC. The content is very reflective of the practices we aim to instill in our students through the libraries, especially topics around structured Markdown notes, Git version control, and notebooks.

 Thank you for also sharing your survey. We hope to learn further about reproducibility challenges, especially with high performance computing, and apply this to further efforts. We are especially grateful that we could receive your comments, seeing that you authored such a thorough and extensive survey on the topic. We will also include this reference in the manuscript.

*“One detail: In the PDF version of the paper, the abstract text is not justified. Shouldn’t it be?”*

**Response: **Thanks for asking this. We will redirect this to the editor
